# Complete mitochondrial genome of broadbanded cardinalfish (*Ostorhinchus fasciatus*) and phylogenetic analysis

**DOI:** 10.1080/23802359.2019.1704190

**Published:** 2020-01-07

**Authors:** Fan Da, Zheng-Yong Wen

**Affiliations:** a College of Life Science, Neijiang Normal University, Neijiang, China;; b Key Laboratory of Freshwater Fish Reproduction and Development, Ministry of Education Laboratory of Aquatic Science of Chongqing, School of Life Sciences, Southwest University, Chongqing, China;; c Key Laboratory of Sichuan Province for Fishes Conservation and Utilization in the Upper Reaches of the Yangtze River, Neijiang Normal University, Neijiang, China;; d BGI Education Center, University of Chinese Academy of Sciences, Shenzhen, China;

**Keywords:** *Ostorhinchus fasciatus*, mitochondrial genome, phylogenetic analysis

## Abstract

In this study, the complete mitochondrial genome (mitogenome) of *Ostorhinchus fasciatus* was first determined and its phylogenetic position was investigated. The mitogenome was 16568 bp long and showed a typical teleost orders, containing 13 protein-coding genes (PCGs), 2 ribosome RNA genes (rRNAs), 22 transfer RNA genes (tRNAs), and a D-loop region. The overall nucleotide composition included A, 25.89%; C, 30.40%; G, 17.46%; and T, 26.26%. Except for *nad6* was located on the light strand, the other PCGs were encoded on the heavy strand. Phylogenetic analysis suggested that *O. fasciatus* shared a close relationship with *Sphaeramia orbicularis* and *Pterapogon kauderni*.

The broadbanded cardinalfish (*Ostorhinchus fasciatus*) belongs to family Apogonidae, order Perciformes, which is a small-scale economic fish widely distributed across the Indo-West Pacific and its adjacent areas (Turan et al. 2010). This kind of fish usually inhabits reef or near-reef habitats, and occasionally be found in estuaries or streams (Thacker and Roje 2009). Although it is widely distributed, researches related to this species are really rare and the exact taxon position of this species is still unclear.

Herein, we for the first time sequenced the mitogenome of *O. fasciatus* by the high-throughout sequencing, and subsequently inferred its phylogenetic position using these genomic data. The samples were collected from Shenzhen city in Guangdong province of China (114°23′68″E, 22°55′70″N), and a specimen (Te-Of2019-01) was stored at Neijiang Normal University.

The full-length mitogenome of *O. fasciatus* was 16,568 bp long (GenBank: MN728946), which was similar to those of that in cardinalfishes including *O. fleurieu* (Zhu et al. 2019) and *Cheilodipterus quinquelineatus* (Matias and Hereward 2018). The overall nucleotide composition of the mitogenome was 25.89% A, 30.40% C, 17.46% G, and 26.26% T, and the mitogenome contained 13 PCGs, 22 tRNAs, 2 rRNAs and a D-loop region. Meanwhile, the mitogenome showed a typical teleost mitochondrial gene orders, which was similar to some other Perciformes fishes such as *Channa siamensis* (Li et al. 2019) and *Channa gachua* (He et al. 2019). The length of tRNAs varied, ranging from 66 bp (*tRNA^Cys^*) to 74 bp (*tRNA^Leu^*). Moreover, three initiation codons (ATG, GTG, and ATA) and five termination codons (TAA, TAG, AGA, T–, and TA-) were identified, respectively. Furthermore, except for *nad6* was encoded on the light strand, all the other PCGs were encoded on the heavy strand.

Phylogenetic analysis was performed to infer the taxon position of the *O. fasciatus*. The maximum likelihood tree was constructed based on a protein dataset of 13 PCGs, and *Beaufortia kweichowensis* was selected as an outgroup (Wen et al. 2017). As found in Figure 1, the tree was divided into two clades of families Apogonidae and Channidae, and the *O. fasciatus* was clustered into the Apogonidae clade and shared a close relationship with *Sphaeramia orbicularis* and *Pterapogon kauderni*. These findings are helpful for better understanding the phylogenetic status of *O. fasciatus*, as well as doing genetic researches in the future.

**Figure 1.**
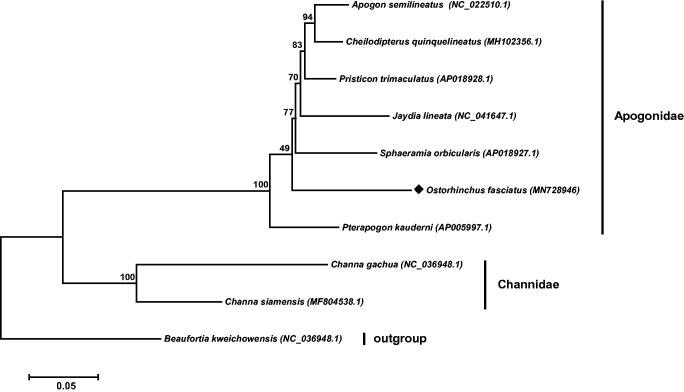
Phylogenetic analysis infers the evolutionary relationship of *O. fasciatus*. The tree was constructed based on Maximum Likelihood method using Mega 6.0 software. *O. fasciatus* was highlighted by rhombic stone, and the *Beaufortia kweichowensis* was used as the outgroup.
